# Characterisation and causal model of the holistic dynamics of the integral sustainability of the agri-food system

**DOI:** 10.1371/journal.pone.0305743

**Published:** 2024-06-27

**Authors:** Luvis P. Leon-Romero, Francisco Zamora-Polo, Amalia Luque-Sendra, Mario Aguilar-Fernández, Misaela Francisco-Márquez

**Affiliations:** 1 Departamento de Ingeniería del Diseño, Escuela Politécnica Superior, Universidad de Sevilla, Virgen de África, Sevilla, España; 2 Instituto Politécnico Nacional, Unidad Profesional Interdisciplinaria de Ingeniería y Ciencias Sociales y Administrativas, México City, México; Taylor’s University - Lakeside Campus: Taylor’s University, MALAYSIA

## Abstract

The transformation of the food and industrial agricultural production system into adaptative and sustainable systems capable of being productive within social, environmental, and economic limits is a crucial factor in reducing the risk to food security and to economic growth. However, the analysis structure of the effect of these variables in sustainable environments remains unknown, whereby the technology and processes are considered as variables of the equivalent critical level as those already described. The purpose of this study is to design a model that enables the characterisation of the agri-food sector based on the determination of sustainable variables from a sustainable and integral systemic approach. Tools, such as the viable system model, are employed to analyse the dynamics and generate the balanced scorecard, to which the items of learning and continuous improvement are added. Lastly, the impact of the principles of sustainability versus the variation of sustainability in the agri-food system is revealed, which is useful in determining the appropriate levels to guarantee a balance in the foundations of circularity. From a systemic approach, this model can be adopted by agronomists and scientists to design alternative strategies for the management of food sustainability.

## Introduction

Activities in the agri-food sector can be of major importance are developed due to their contribution to the economy and to human life itself. Hence, there is great interest in studying them from the perspective of holistic dynamics [1], that is, from the changes and interactions that arise across the entire sector, which comprise the flow of people, information, energy, and materials, among other structures and organisations. Through these activities, issues of economic, social, environmental, and technological utility emerge from the execution of the processes demanded, and these affect the health, well-being, and balance of the entire local, regional, national, and international ecosystem [2,3].

However, regarding productive activity, human beings exploit the land and other resources which causes adverse effects; these in turn affects the environment, economy, and society. Nevertheless, this production is achieved through the design, creation, and implementation of processes and technologies as tools to facilitate operations. To this end, this research considers it is important to bear these factors in mind when discussing and assessing the sustainability of the sector. The space occupied by crops represents 37% of the arable land surface, and the use of water for these corresponds to almost 2/3 of the total area of arable land [4,5]. One of the effects on the environment that of pollution by nitrates, phosphates, and pesticides, which act as a source of production of greenhouse gases, methane, and nitrous oxide which affect the quality of both air and water [6,7].

Poor land management leads to stress which produces degradation by salinisation, excess water extraction, and contamination of groundwater by agrochemical residues. This is due to the use of excessive quantities by crops and to the generation of an imbalance in the ecosystem, which in turn affects the environment, productive capacity, economy, and society [8–10].

According to Benabderrazik et al., food security and economic growth in the regions should be associated with a balanced interaction between social and economic well-being and the protection of natural resources, since an intensification of activities in these sectors can lead to unsustainability, which puts the general well-being of the population and the productivity of the region at risk and affects the ecological environment. These components interact in a non-linear, complex, and dynamic way, and it is therefore pertinent to analyse the feedback received by the system to offer sustainable and adaptable solutions to environment situations[11].

The theory of adaptive systems represents an effective framework for the analysis of the dynamics of systems in terms of their transitions. Since cyclical evolution seeks equilibrium when changes arise, the agri-food sector articulates a type of complex adaptive system in which there is an exchange of matter, energy, and information both internally and with external structures, through the ability to self-manage, which corresponds to its systemic functionality [12].

The incorporation of strategies, such as life cycle analysis (LCA), enables the development of products in a conscious way regarding the impacts generated in each stage, from design to final disposal. This leads to reasonable agri-food production, and improves their environmental, economic, health, and well-being properties with the help of technologies that contribute towards this end. The levels of efficiency and productivity of the processes are also improved by making better use of the resources; this will have a positive impact on the accessibility to resources for future generations [13,14].

A considerable reduction in waste generation is expected through the application of the circular economy from the perspective of reducing, reusing, and recycling in work models, together with the influence of eco-design and the use of raw materials from renewable sources. This is good for society, the environment, the economy, and the efficiency of processes, since it guarantees the sustainability of operations from the management of technology and information [15–17].

It is worth considering the benefits of increasing our capacity to generate cleaner energy that is more sustainable by transforming waste that cannot be reused or recycled. When we subject waste to treatment in order to reduce its volume and produce energy while reducing dependence on fossil fuels, it leads to better waste management with positive impacts on society, the environment, technology, the economy, and the overall process, and hence affirms the sustainable nature of the system. This has been demonstrated in previous research and has highlighted the energy valuation of waste as a benefit of the circular economy practice in agri-food processes in that it reduces environmental emissions and increases energy efficiency [18–27].

This article aims to characterise causal model holistic dynamics from an integral sustainability approach regarding the agri-food system. The processes and technology are integrated into the traditional model of sustainability in order to strengthen and promote a holistic analysis for the generation of suitable management strategies towards the improvement and continuous learning of the system in such a way that it contributes to its co-evolution.

Therefore, the following research question is proposed: does intervening in the current sustainability model by including additional process and technology variables allow for a holistic analysis of the agro-industrial sector in order to provide integral strategies for the sustainable co-evolution of the sector? As a complex adaptive system, it is useful to identify the elements and relationships that make up the system, then graph the dynamics while considering the interconnections of the variables, and subsequently to analyse from the model of viable systems for the conclusion of a balanced scorecard that serves as a support tool for the management of the improvement and learning of the system, that is, the evolution of the system towards the set of objectives.

## Literature review

The agri-food sector, as the integration of the production, distribution, and consumption of food, is one of the sectors most strongly affected by the crisis of climate imbalance [5], and by unfavourable practices in its operation which alter its holistic dynamics [28]. This exerts a direct impact on sustainability, and therefore reduces the opportunity to guarantee better levels of health, a better supply of food, a better environment and an efficient level of water, air, and soil for future generations. However, it is the agro-industrial sector that can most help improve conditions regarding environmental problems that have already become a concern today, by making use of best practices in its basic operations [29,30]. In Europe, research and development are being carried out to increase the value of unsuitable food materials unsuitable for human consumption (wrappings) and to consider food waste and residues, by focusing on economic and environmental variables [31].

Riemens et al., focused their research on the holistic improvement of herbicide chemicals to increase the sustainability of the agricultural sector, since these chemicals are critical in the management of weeds that compete with crops for resources such as light, water, nutrients, and visible space, and can affect crop yield the environment, and human health [32].

Water scarcity is another factor that conditions the integral sustainability of the agri-food system, since the use of water resources without considering any of the effects can that lead to socio-economic problems and degradation of resources; it is therefore necessary to study the underlying variables in the agri-food system to prevent these unforeseen effects [11].

Water, a crucial resource, is being affected by climate change. The rising temperatures have caused a reduction in rainfall and increased drought, leading to the scarcity of water. This uncertainty is alarming for the agri-food sector as water availability is closely linked to production and quality crop yields. Effective management is essential for the promotion adaptability, and sustainability of the system, by ensuring that food security, and the economic, social, and environmental conditions are preserved. Certain processes and technologies must be considered in order to achieve this goal since not only do the hydrological cycle and surface energy balance vary across different types of land use, but vegetation also influences temperature, due to regulation by heat exchange, which in turn affects agricultural development and natural ecosystem [33–42].

On the other hand, substantial rainfall over short periods of time produces overflows which carry particles that can pollute bodies of water, such as rivers, which put at risk the quality and productivity of the activity of the agri-food sector. Other aspects involve the wells of illegal underground aquifers that divert the course of the aquifer sources and contaminate such sources through their mismanagement, which highlights the crisis of governance in the sector [43].

Diogo et al., highlighted the importance of the social component within the sustainability model; in their research, they demonstrated that the social component is the least developed element and that to date, no evaluation tool has enabled us to recognise the dynamic interaction of ecosystems with communities [44].

On the other hand, Cook et al., argue that expansionism has dehumanised those agricultural activities influenced by power and the desire to improve productivity by ignoring the significant contribution of farmers, who must either adapt to new technologies or give up their work. The authors point this out as unfair from the social point of view, and also recognise that change in complex systems must include commitment to the existing members who carry out the day-to-day practices in that system. Furthermore, they state that the work by women is minimised in the implementation of the expansionist concept and that the results in terms of the productivity of the sector and the welfare of farmers are not evident [45].

However, studies such as [46] consider expansionism as a tool that can enable the fulfilment of SDG 2 of the United Nations, wherein it is projected to end extreme poverty and hunger. These authors also consider that this strategy of transfer of knowledge and technology to farmers can contribute towards the solution of problems specific to the work involved and the environment in which it takes place, but they also argue that it is necessary to shift attention away from specific behavioural changes and move it towards the creation of awareness, with which learning becomes more productive. Furthermore, they state that sustainability, behavioural change, and technology adoption are less relevant than is the increase in farmers’ self-confidence by engaging and playing a significant role in the agricultural development process.

Studies have been carried out to analyse the elements that obstruct the ability to exercise practices that contribute towards nature conservation. These studies are based on innovation systems approaches, with emphasis on the development of multiscale frameworks that are specific to each environment and relevant to the integrated evaluation of sustainability of changes in agricultural intensity [47].

The tools available for the application of systems that are linked to the circular economy are largely aimed at major companies, and exclude SMEs, which to a lesser extent, are also aware of the need for the transformation of their production systems regarding the general welfare of society, the economy of the regions, and the conservation of the environment. However, Industry 4.0 is already offering sustainable practices focused on the circular economy in these organisations [48,49].

Regarding the life cycle in the agri-food sector, the processes are given by two components: one biological and the other technical [50]. The former is capable of being reintegrated into the biosphere with which it is intended to create alternatives of waste instead of accessing processes of biochemical extraction, composting, and reincorporation into the biosphere, while the latter is destined to be revalued without entering the biosphere, since at the end of its useful life they could be repaired, reused, or recycled to obviate the need for the extraction of new raw materials [51,52].

It is necessary from the moment of design to consider the elements that form part of this product and the appropriate technologies to minimise its impact. This impact can be in many forms: in terms of abiotic resources, acidification, eutrophication, global warming, depletion of the ozone layer, human toxicity, ecotoxicity in fresh water and soil, and the formation of photochemical oxidants. All these impacts bring an imbalance into the ecosystems which in turn can affect the environment, health, and society, with economic impacts for their repair. The efficiency of production processes in general of the agro-industrial sector can also be affected, mainly those of a biological nature [7].

In the absence of the efficient design of sustainable processes and products under the framework of circularity, there are aggravating situations, such as that of food waste, which currently accounts for a third of total food production. It is estimated that edible food waste in the European Union is approximately 89 million tons each year, of which 42% is produced by households, 39% by food manufacturing processes, 14% by catering services, and 5% by the distribution sector [52].

Product Lifecycle Management (PLM) as a tool for managing the life cycle of a product allows the visualisation of the development and progress in projects of the creation of products and services, in order to previously ascertain the costs and other information of interest of said products, thereby attaining a better view of the best options, in order to make efficient decisions without interfering negatively with the environment. This tool facilitates the integration of project stakeholders by making information and traceability available at each stage of its development [31,53–55].

Product Lifecycle Management is a vital tool that covers from product conception to the final disposal of the product [56]. This process occurs in 3 phases: beginning of life, middle of life, and the end of the life of the product itself [57]. Additionally, Salonitis and Stavropoulos, argue that the conventional PLM system only focuses on the first phase, and hence suggest an extension to the other phases. The recognition of other limitations highlights not only the integration of different tools on a digital platform, but also the interoperability of both internal and external systems and devices, and the number of files and data exchanges between stakeholders in all phases of the life cycle [56].

On the other hand, PLM contributes towards the transition of Industry 4.0 from the digitalisation of processes to better control, by monitoring via good traceability and follow-up practices for better decision making. In addition to Industry 4.0 tools, such as artificial intelligence, IoT, machine learning, and big data, PLM should be integrated into the agri-food sector and precision agriculture to optimise productivity and resource efficiency [49,58–60].

Life cycle analysis and the circular economy lead to cleaner production spaces: LCA contributes towards the quantification of the environmental impacts present in the study system [61], while the circular economy helps to identify and define the scenarios to improve the characteristics of such a system [62]. This is an iterative process that allows the measurement of good practices and their real effects [15,61]. However, authors such as Poponi et al., argue that LCA study proposals have been criticised for not being suitable for specific products, since it is a methodology designed for products at only a general level [63].

In general, the processes focus on meeting the United Nations Sustainable Development Goals (SDGs), in which SDG 12 is aimed at ensuring sustainable consumption and production patterns and includes targets that aim to achieve a more efficient use of resources [64], Therefore, the management and analysis of the life cycle is of major assistance because they lead to this compliance from the early stages, such as design, until the product is no longer in a useful life phase [65].

Thanks to technological advances, there are tools, such as specialised analysis software, that are easier to perform due to their complexity. Life cycle analysis is included in several of these programs: some require payment while others remain free and multiplatform. These include Air.e LCA, Open LCA, SimaPro, and Eco-it, which are helpful since they lead to clarity on the levels of climate change, human toxicity, ecotoxicity, and depletion of biotic resource information of interest, to facilitate decision-making within the development of products [13].

In general, the relevance of tools such as management and life cycle analysis on the sustainable approach and circularity are necessary due to the estimates made for 2050, the year in which there is a predicted increase of between 60 and 70% in food consumption, which compromises the resources available today. Water consumption, for example, it must increase by 30%, while energy demand is expected to rise by 45%. However, the transport sector has the most representation among the registered carbon footprints, with 18% of the total. The transport of food products accounts for between 15 and 30% of the carbon footprint of the food and beverage industry, given that this is a great ally for the distribution, mobility, and accessibility of raw materials, inputs, final products, people, and machines, among the many goals that also form part of the agri-food sector [52,66,67].

Several studies show the importance of the integration of technology and processes regarding sustainability, which is why it is necessary to modify the traditional sustainable triad made up of the economics, socials, and environments factors. In order to achieve an expansion towards the sustainable pentagon where the technology and process variables are considered, it is necessary to focus on providing better attention and intervention in these factors. This focus can lead to a holistic and integral analysis of sustainability, which is relevant for the creation of strategies, for policies, and for decision-making in favour of the sector [68–72].

The best way to face complex problems, such as determining the sustainability of a system, is through system dynamics, a systemic approach, and systemic thinking in general, since these problems are complex and their behaviours is not linear, as a consequence of the feedback loops of the interconnections of the many variables of which these systems are comprised. Various authors have employed tools, such as causal loop analysis, to understand the systemic dynamics of the systems under study and to establish possible solutions to support improvement [73–76].

## Materials and methods

**Fig 1:**
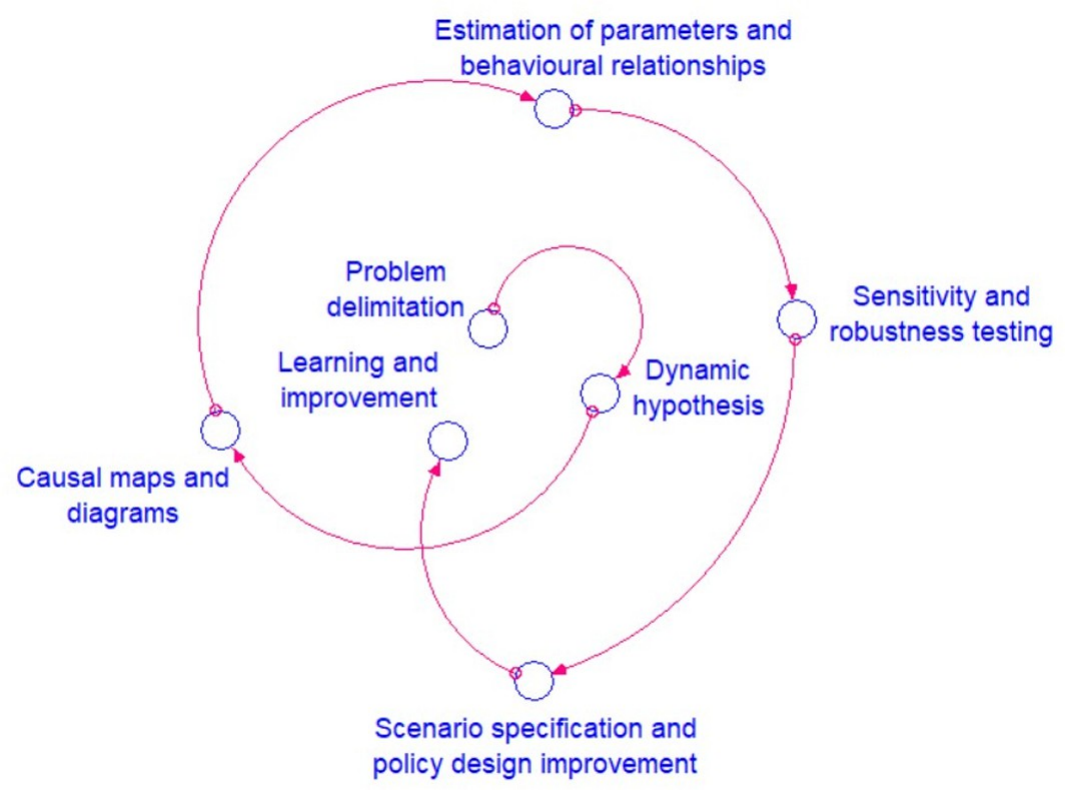
Methodology considering Sterman’s essential steps taken from [78].

This work was developed using qualitative and descriptive research within the framework of dynamics, and systemic thinking. The methodology used is based on Sterman’s model [77], which is described in detail in [78]. The implementation process is illustrated in Fig 1 and begins with the identification of a problematic situation in the field of study and draws on the contributions of Peter Checkland [79,80]. This process acknowledges the gap between perception and reality and aims to address this difference in order to achieve an ideal outcome.

Clarity regarding the variables is necessary for the design of improvements to the system by creating purposeful systemic models where those variables are interrelated.

Causal maps and supporting diagrams are created to reflect the interconnections, for which the model of viable systems proposed by Beer [81,82] is also utilised, from which parameters and estimates of behaviour of the system of the variables are established. Thanks to the modelling, the results can be analysed and yield a knowledge of the dynamics of the system which enables performance of this system to be acted upon. Through of the application of the methodology proposed by Kaplan and Norton [83] for the creation of the balanced scorecard, favourable and viable changes are estimated according to the specifications of scenarios and the design of improvement policies, which leads to a new reality of the system that will again be contrasted with problematic situations perceived in this new phase. This methodological process will be applied iteratively to generate knowledge, learning and a continuous improvement of the system under study.

For the characterisation of the sustainable holistic dynamics of the sector, it was necessary to define the variables of interest that constituted the pentagon of integral sustainability, that is, the five main variables for this model. Subsequently, an evaluation of influence between these variables is carried out, that is, in a Likert range of 1 to 5 (with 1 indicating a mild effect and 5 strong effects) with each of these variables influencing the others. The points awarded are added for each variable, and the highest result indicates the variable that receives the greatest influence from the remaining variables which renders it the most affected of the system. The characterisation of this variables is then carried out for its subsequent analysis within the system under study while considering the other variables of interest of the model.

With this evaluation, it is also possible to ascertain which variables exert the strongest effects on the others, while recognising the role of systemic analysis that allows connections and integration without ruling out the uniqueness of each variable so that a better understanding of the system under study can be achieved [84].

Initially, a systemic causal model of the agro-industrial sector is created through the Ithink 8.0 modelling software. This enables the diagramming of the model of viable systems to proceed with greater precision, and to analyse the relationships of the subsystems that make up the system under study. By considering the external environment, the information is obtained for the creation of the balanced scorecard in order to identify the variables of interest of the sector from the perspective of integral sustainability. The mission, vision, objectives, indicators, goals, and actions to be carried out are all established. Furthermore, the following fields are also integrated: learning, short-term improvement, and long-term improvement. These last three items are necessary when the goals and the result of the indicators reach a level of deviation greater than 5%. Their aim is to self-regulate the system so that the established objectives remain unaffected, which enables a culture of learning and continuous improvement to be attained, given that this is an iterative process.

Lastly, five basic principles are postulated to guarantee integral holistic sustainability, and these are analysed from their link to the process studying their effects on the variables that constitute the pentagon of integral sustainability of the proposed model.

## Results

### Identification of variables of interest

**Fig 2:**
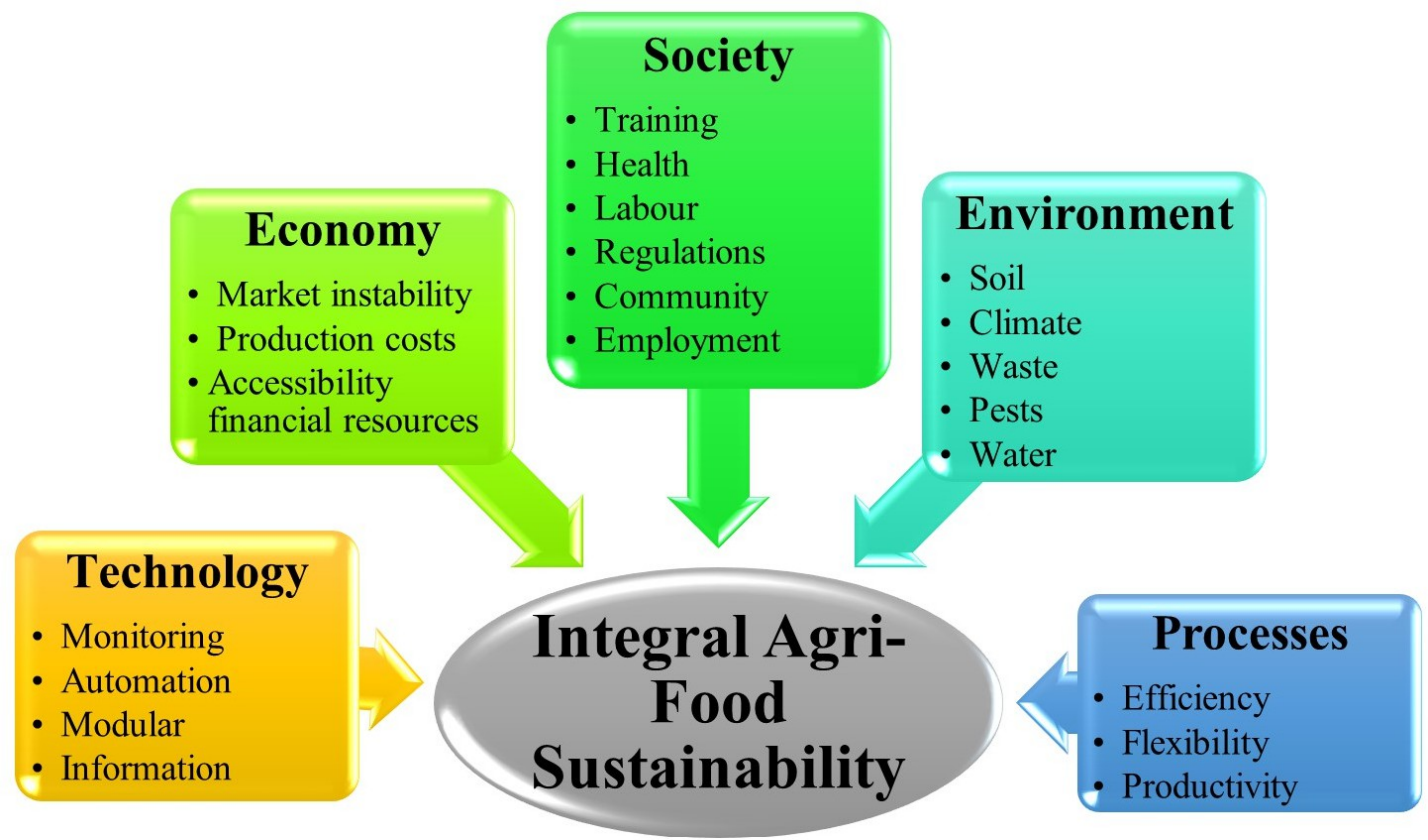
Elements of the agrifood integral sustainability model.

The model of sustainable integration in the agri-food sector is given by the many variables that exert an impact on the fundamental categories or their macro variables. These include the traditional Economy, Society, and Environment factors; in this work, Technology and Processes are also considered. As showing in Fig 2, each of the above factors contains different variables that define them within the sector under study and distinguishes the sustainable integration model.

**Fig 3:**
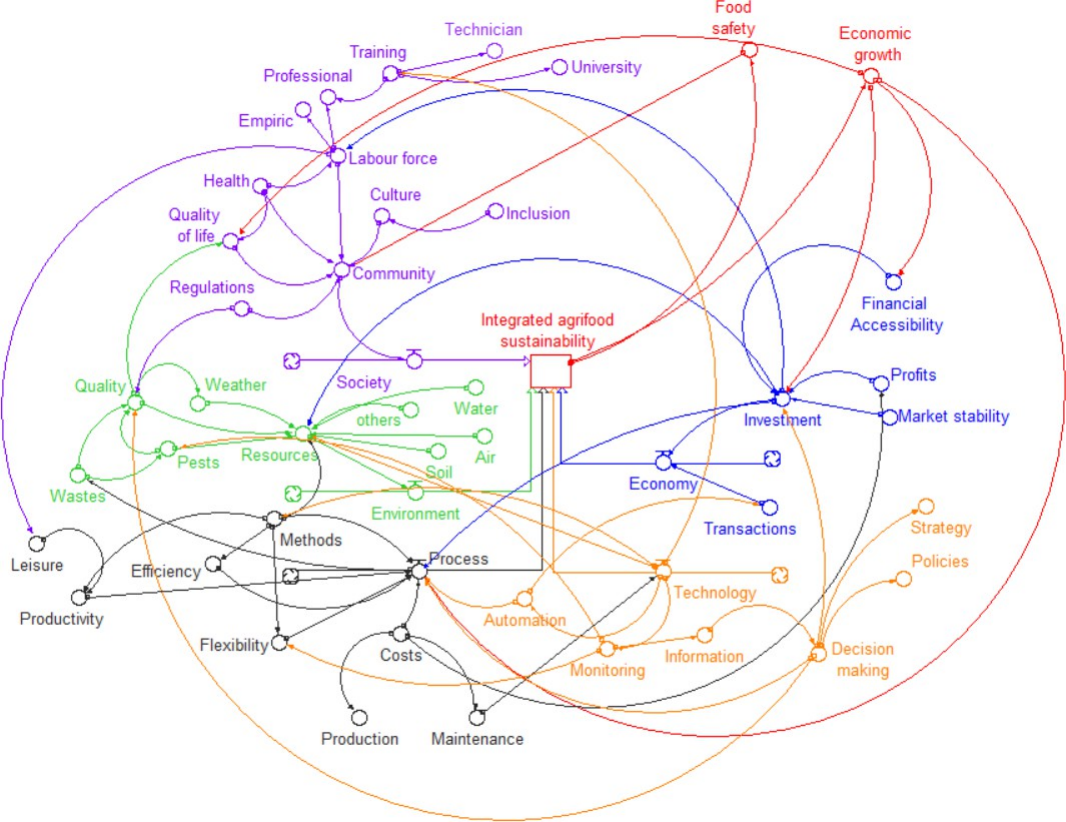
Causal map of integral sustainability variables.

**Fig 4:**
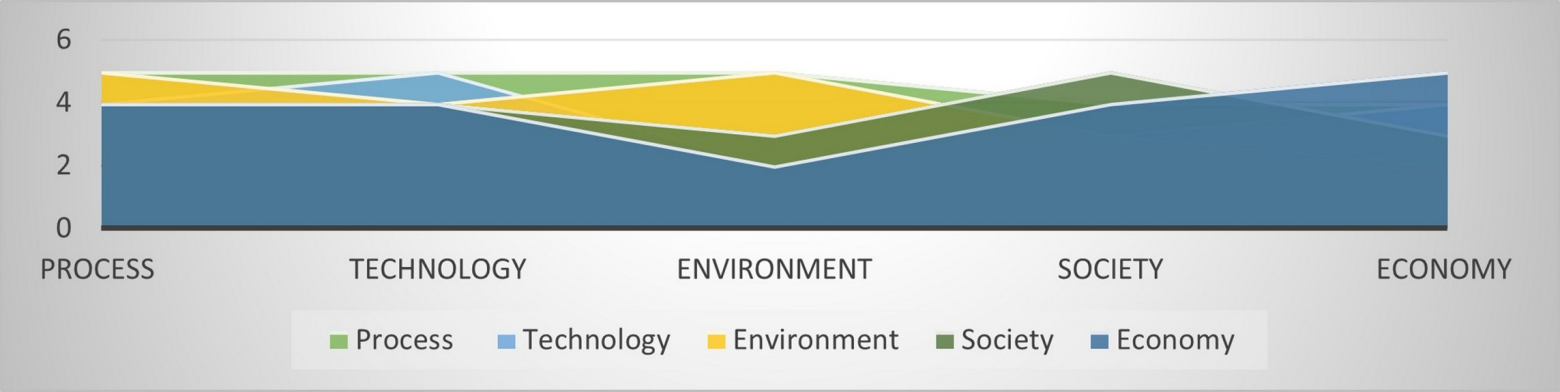
Influence of integral sustainability variables on each other.

The relationships between the variables starting from the conception of a general systemic approach are perceived from the way they interact and produce effect, and hence the need to achieve a balance that allows the harmonious functioning of the system. Fig 3 shows the causal map, and the Fig 4 shows the level of influence between the technology, process, environment, society, and economy variables as the main variables of the integral sustainable system model in the agri-food sector.

In the Fig 3, it can be observed that, in this case, the variable that receives the greatest influence from the others process variable, and the variables that most influence the others are: process, technology, and economy. Table 1 shows the values of such an evaluation.

**Table 1:** Level of interaction and influence between the main variables of integral sustainability in the agri-food sector.

Integral Sustainability Variables	Process	Technology	Environment	Society	Economy	Variable that most affects
**Process**	5	4	5	4	4	**22**
**Technology**	5	5	4	4	4	**22**
**Environment**	5	2	5	3	2	**17**
**Society**	4	3	3	5	4	**19**
**Economy**	4	4	2	3	5	**18**
**Variable that is most affected**	**23**	**18**	**19**	**19**	**19**	

The process variable, with a score of 23, is the which most affects the others: in order of influence, this is technology, economy, environment, and society. On the other hand, those variables that have the most impact on the other variables with a score of 22 are technology and process. (S**ee S1 Appendix**).

### Integral sustainable holistic dynamics of the variable process in the agro-industrial sector

It is possible to have two divisions of the materials that circulate in the processes, one biological and the other technical Cañoles et al., first referred to the benefit of organic matter produced along the food production chain, from primary production to consumption, and to the various types of organic waste that is generated, such as sludge, agro-industrial by products, inedible food remains, and food waste [50].

In contrast, technical materials are related to the use of machinery, plastics, and other industrial elements utilised in agricultural production.

**Fig 5:**
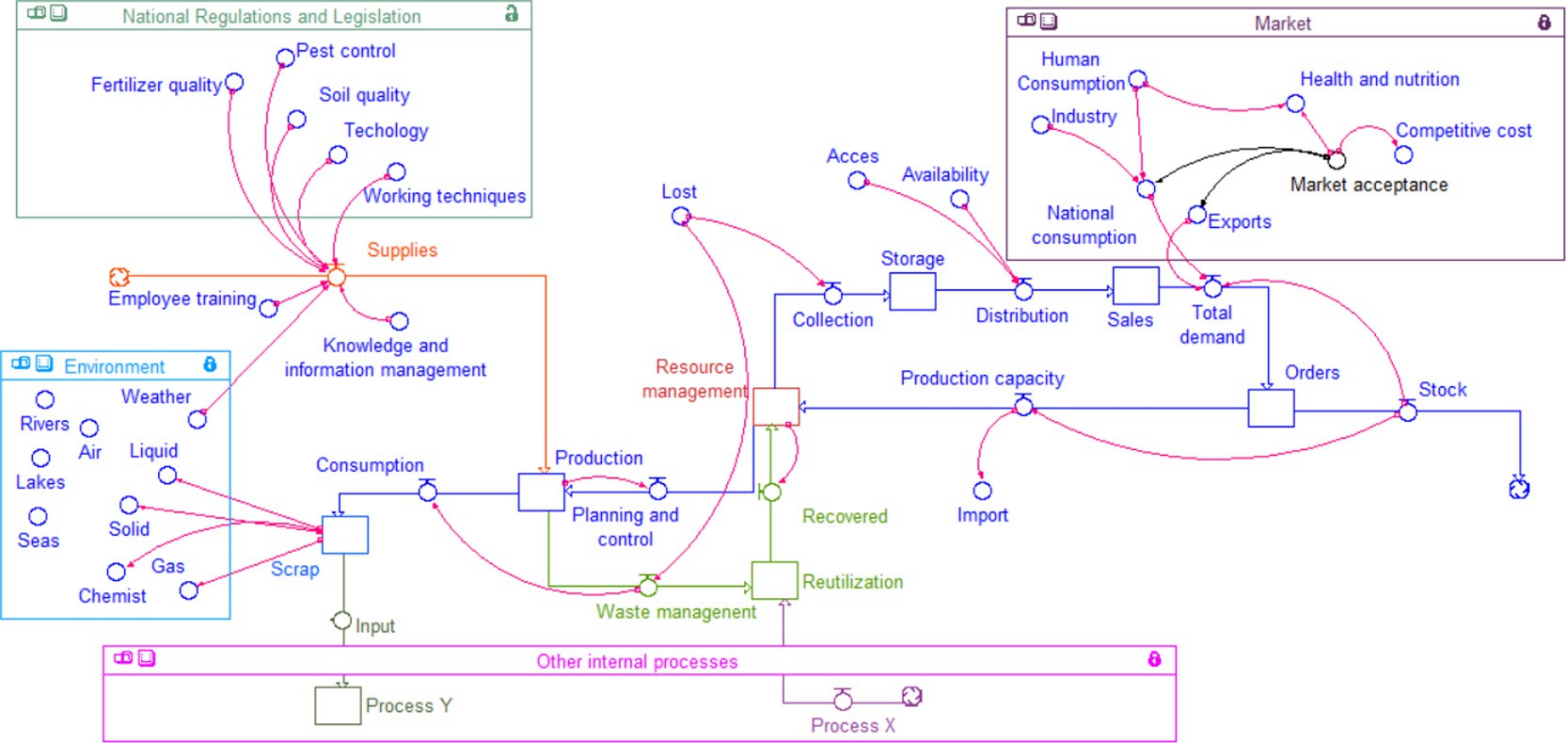
Systemic model of sustainable integration in the agri-food sector.

From the Forrester model represented in Fig 5, the relationship of the variables that are part of the process in general are show, whereby the biological and technical aspects of the agri-food sector are integrated.

The model integrates the internal production variables, from the management of resources where the planning and production control are considered starting from the demand, and the capacity of the process, to deciding whether to import and/or proceed to produce, while considering waste management, reuse, and recovery of materials, all integrated with other alternative processes. Furthermore, the model includes the variables that are affected by the waste generated, such as water sources, soil, air, and the climate present in the environment which can be internal or external. In same way, this model reflects the influence of the standards, legislation, and current trends in including the of quality of fertilisers, pest control, and the quality of soil for planting, which affect the supply of inputs and/or raw materials. Lastly, there is the influence of market dynamics (economy and society) on demand.

### Holistic and adaptive dynamics of the agro-industrial sector based on Beer’s viable model and the integration of the balanced scorecard as an iterative process that contributes towards learning and continuous improvement of the agro-industrial sector

**Fig 6:**
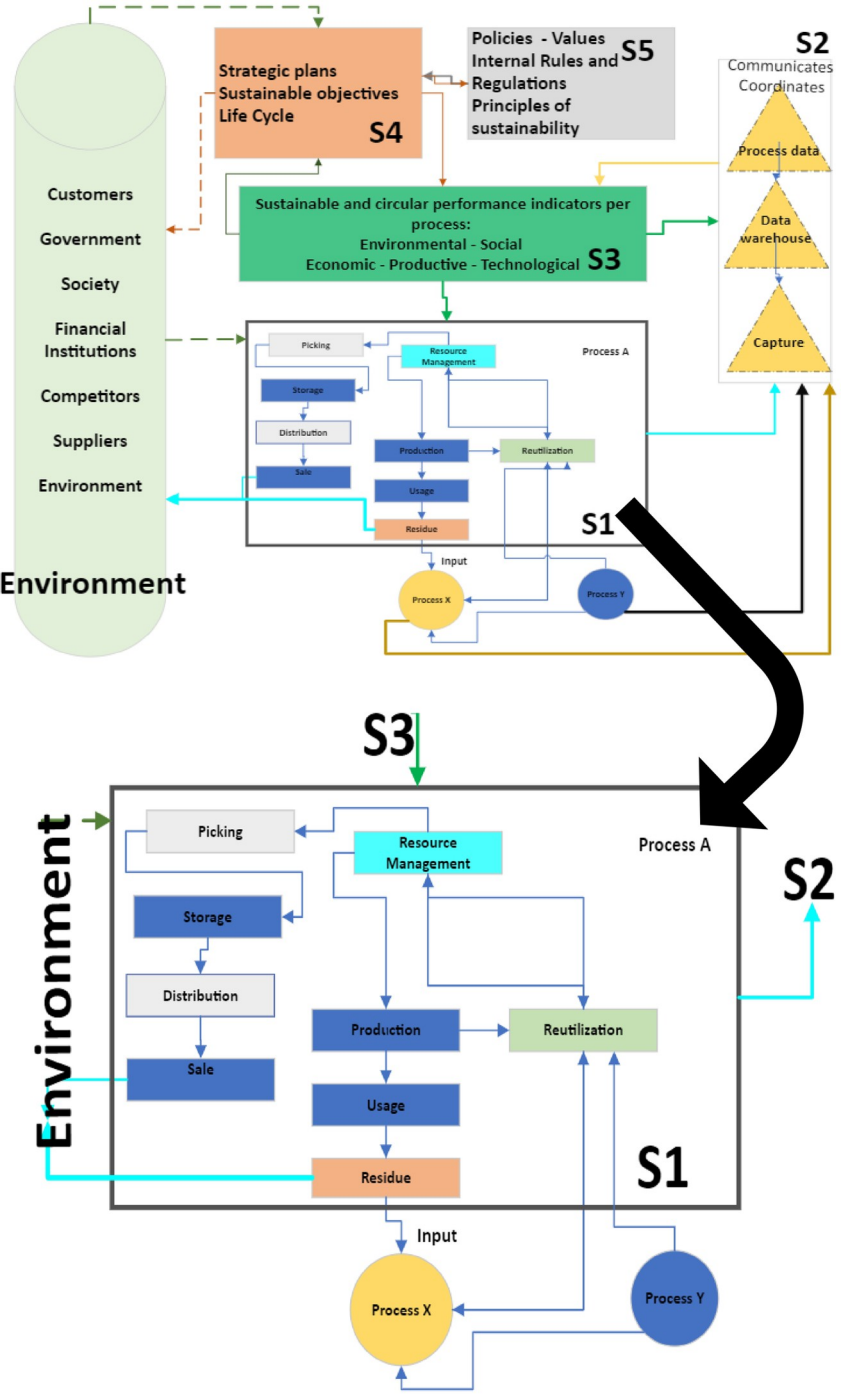
Model of viable systems based on Beer´s model (S1 = System 1, S2 = System 2, etc).

Fig 6 represents the model of viable systems, which is divided into 5 internal systems that are related to each other and to the external environment. From System 5, policies are created for the conservation and improvement of environmental, social, productive, technological, and economic aspects as the main categories of the holistic system of integral sustainability. Based both on the policies, ethics, and values that characterise the sector, and on the work regulations as a regulatory framework for strategies and plans in accordance with the mission and vision, this system has a direct relationship with System 4. System 5 establishes the categories of interest of the scorecard (see Table 2).

**Table 2:** Balanced scorecard designed from the viable system model of the sector under study.

Category (S5)	Objective (S4)	Indicator (S2)	Meta (S3)	Initiative (S1)
**Society**				
**Environment**				
**Economy**				
**Process**				
**Technology**				
**Learning:**				
**Immediate S1 improvements**				
**Future improvements S4**				

In System 4, the long-term work plans, and the objectives presented in the balanced scorecard are projected regarding the entire life cycle, which is necessary to meet the estimated goals given the aspects that System 5 safeguards. Therefore, each category established in System 5 is supported by several objectives for its conservation and improvement as the fundamental pillars of the system.

System 4 feeds and is fed from two inputs, one internal input determined by System 3 and an external input represented by the environment. From System 3, the information of the deviations or internal performance of the system in general is received, that is, everything that requires future improvement is communicated to System 4 so that it can create the relevant strategic structure for an efficient result. Once this structure is established, they are again transferred to System 3 and hence they can be lowered or delivered as input to System 1, in which the basic procedures and tasks are executed so that the system flows and continues its operation.

From the external environment, System 4 meets all the requirements of the client, government, and environment among others, and considers regulations and trends, which enables better preparation towards offering answers and adaptation to the changes that the future holds in terms of sustainability variables, thereby reducing the levels of uncertainty in term of the future.

Returning to System 3, this is responsible for the monitoring, control, and measurement of the internal system; the established goals or parameters are found therein and must all be met to ensure the proper functioning and viability of the system. The goals are included for each objective in each category regarding the variables of integral sustainability namely, economy, society, environment, process, and technology.

This system is supplied by the flow of information from System 2; such information is compared with the value of the estimated goal allowing us to calculate and observe the degree of deviation of the real results of the system from its ideals.

The results of the deviations are averaged per category and plotted according to the Pentagon’s sustainability; the categories in the graph that indicate a deviation over 5% will require an analysis for their prompt performance improvement, and it will be necessary to create a graph for each category individually. With the graphs of the independent categories, the indicator that requires the most attention can clearly be detected. If it is an immediate action for its repair, then System 3 must be connected to System 1 for its performance; if it is an improvement that needs planned for future effect, then this must be communicated it to System 4. This control exercised by System 3 will reveal the real health of the system compared to its ideals. It is of major impact to carry out the necessary actions so that the system follows the course as planned, which also contributes to its self-regulation.

System 2 is the one which transmits the information to System 3 and receives the results of the operations carried out in System 1. Each objective described in System 4 is assigned a metric or calculation formula to ascertain the level of achievement of these operations, which is established in System 2. The data to activate the formula or indicator correspond to the results of the tasks or activities developed in System 1.

By capturing the information of the executed processes, the data is stored in System 2, where the operation is carried out according to the established indicator or metric whose result provides a real indication of the behaviour of the system based on the evaluated variables of integral sustainability. Once captured, stored, and processed through calculations already programmed the real results of the operations already executed are communicated to System 3 to proceed with the comparison in accordance whit the goals. The information in System 2 is always available in up-to-date or historical form for future reference.

System 1 carries out all the basic operations and implementation of the initiatives necessary for the system to function in general. These operations correspond to the biological and technical processes of the sector given by the supply of raw materials, resource management, production, reuse, collection, distribution, training processes, inclusion according to requests, and requirements received according to the variables of the integral sustainability of the sector. The result are then transmitted to System 2.

System 1 carries out the activities that were planned in System 4 and transmitted by System 3, in addition to the interventions of the environment that require immediate action. Consequently, the environment has a direct effect on Systems 4 and 1 in the same way as these systems interact directly with the external environment.

### Organic principles of the holistic systemic causal model of integral circular sustainability in the agri-food sector

**Fig 7:**
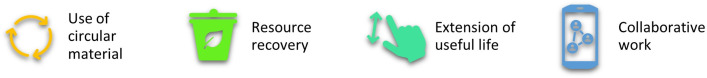
Basic principles of the sustainable model.

The sustainable model is given by four principles, as illustrated in Fig 7, which facilitate the circularity of the materials and inputs used in the production process of the agri-food sector. These allows interaction with other processes and the collaboration of stakeholders in the sector as responsible for the work of minimising negative impacts to the environment that affect society, the process itself, and the economy. It is recognised that technologies must also be developed for this effect since they affect the variable and are not affected by said variable.

**Fig 8:**
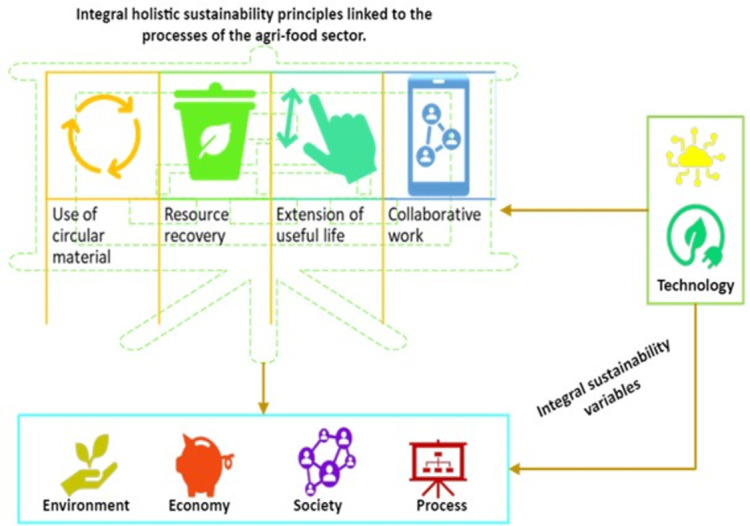
Basic principles of the sustainable model integrated into the process.

Fig 8 represents a diagram in which the influence of the principles is observed once they are linked to the process. Herein, it is possible to consider that they create an effect on the categories of environment, economy, society, and on the process itself. Nevertheless, it is technology which affects the principles, and hence they must be adapted or in favour of them.

## Discussion

Although the environments, society, and economy variables are those that by default are considered previous to the studies of circularity and sustainability, it is currently a framework that must expand and integrate other variables that also require equal importance in the face of these analyses. To this end, technology and process variables are added, whereby on the latter the life cycle of the products is considered from the early stages of their creation to their final disposal.

Scientific evidence justifies the impact of technology and processes on sustainability results. This identification leads to a positive relationship between the inclusion of these variables in the traditional triad, thereby making a holistic and comprehensive analysis possible in the sector [22,85–94]. However, there is no formal information on this inclusion in any such analyses, possibly because it is implicit within the model, and hence the three common variables, namely society, economy, and environment, always prevail.

Technology is of major importance [49] due to its implication for the development of activities that trigger major consequences in the events that are executed in the sector under study that put their sustainability at risk. Furthermore, the processes leave traces that alter the life cycles within a region if they are not studied in a systemic way, whereby their effects in the environment where they develop, such a population and the very existence of the process are put at risk.

Determination of the variables and ascertaining how they affect each other allows us to attain a broad knowledge of the sector and to detect which variable requires more attention. This study can present a call for help in maintaining the balance while not exposing the integral sustainability of the sector.

The processes of the agri-food sector require attention depending on their nature; some are biologically necessary for the planting and cultivation, while the technical processes contribute to their advanced operations. It is crucial how they flow and how these two variables of the process are integrated, given that they have influences from the external environment from policies, regulations, trends, markets, and from the environment itself. It is also necessary to determine the impacts to which they are exposed and to what degree they are able to influence the internal media must also be considered, considering the internal policies given by the mission, vision, values, and internal regulations that regulate the relationship with other internal processes [52].

This dynamic facilitates the analysis of the operations that need to be restructured and reveals their incidence against the internal or external processes with which they have direct and indirect links, which will ease their coexistence between them from the homeostasis of the system in general [95]. Recognition of the effects on other processes leads to the creation of awareness [46] which that contributes to the competitive and productive permanence of achieving objectives. Remembering to be a teleological process, that is, it has purpose and determination since it is not random, but instead is planned and premeditated [95], and also involves a major impact on the economy, environment, interest groups, the technological means to be used, and on the processes themselves.

Holistic and adaptive dynamics of the agro-industrial sector are based on Beer’s viable model, whereby the balanced scorecard is integrated as an iterative process that contributes to learning and continuous improvement of the agro-industrial sector. Knowledge of the holistic dynamics is an increase in the adaptability, viability, and therefore the sustainability of the sector which renders it more economically, technologically, socially, and environmentally responsible, thereby preserving its integrity for future generations. With good results, it can be improved with the reasonable practices of the resources available, which render it being increasingly productive and efficient [14].

The fusion of systems analysis through Beer´s viable model and the balanced scorecard enables us to conclude the strategic structure in a systemic way, which is relevant for a better operation and achievement of the established objectives. The mission and vision are considered of the system under study integrated in System 5 of the model. In addition to this work, items of interest that contribute towards sustainability are added, such as the learning that is generated, especially when an objective has indicators that fall below the level of the established goal, which helps the approach of new and timely proposals for improvement to develop in the short or long term.

The balanced scorecard designed from the viable system model of the sector under study allows the evaluation of the variables of integral sustainability as an iterative process that contributes towards the learning and continuous improvement of the agro-industrial sector [96]. This interaction leads to the creation of strategies [97] that should be included in the design from its link to the systemic process. To go beyond determining low performance rates and proposing improvements is to recognise the learning regarding what could not be achieved with the resources that were available.

The organic principles described for this model, such as use of circular material, recovery of resources, extension of the useful life of the product, and collaborative work, are all perceived as necessary to guarantee the quality of the integral holistic sustainability of the system. Hence, its integration is of value and in which its interaction with the main sustainability variables must be considered and must be recognised as to which affect the principles, and which in turn are affected by said principles [52,65,98].

In this opportunity, technology holds the key to compliance with the principles, and the use of artificial intelligence is a major contribution toward operations and results of greater efficiency [59]. Therefore, the principles are also considered a restriction for the selection of this, since the application of the principles in the model exerts a direct effect on their performance thereby creating an impact on the other categories of the sustainable pentagon, namely the environmental, social, economic and process variables, which are also affected by technology. These last four do not affect the principles, but rather, they themselves are affected if they are not executed rationally and consciously.

## Conclusion

This characterisation the sector through causal maps, based on the determination of the critical variables in terms of integral holistic sustainability, leads to the creation of a strategic structure from the improvement and continuous learning of the sector as a complex adaptive system. Its value focuses on permitting its location on the sustainable plane as a contribution to current global problems from the systemic use of viable models that enables three fundamental points to be ascertained:

 ○ Determining where the system is today by analysing Systems 1 (basic operations for normal operation) and 2 (historical, current, and actual system information system) of the viable system model. These systems are critical since they form part of the starting point to ascertain the path of improvement that the sector requires according to its objectives.
 ○ Ascertaining the direction to follow constitutes another aspect of value that is estimated in System 3 (control system, audits where the ideal parameters and goals are established). The clarity of the goals is important because they direct the processes of self-regulation of the system in general.
 ○ Ascertaining what is needed to reach that desired goal regarding the integral sustainable performance of the sector so that not only does it works internally but it also has a positive impact and adapts to the external environment. This is achieved in System 4 (a system that allows future strategic planning to better respond to the environment) by considering the conditions and constraints of System 5 (policy system, values, mission, vision that direct the system).

As an iterative process, it introduces performance into the systemic approach of continuous improvement and learning.

This research has concluded that variables process, and technology need to be integrated into the traditional model of sustainability to ensure a more comprehensive and holistic analysis, thereby generating awareness based on the principles of integral sustainability determined in the proposed model.

The process of designing and establishing stable, balanced, and flexible methods, techniques, and timelines for a sector must be carried out while also considering the holistic nature of the system and the resources and technologies available. This is crucial for the sustainable integrity.

Highlighting its goodness, the model represents a neurocybernetic metaphor that mimics the biological functioning of the human body as a biological machine that has evolved over millions of years [99–102]. A further contribution is that the model of viable systems is connected to the balanced scorecard as a reinforced tool, which contributes towards the continuous improvement and learning of the system. This is an iterative evaluation practice designed for the co-evolution and adaptability of the system.

## Limitations and future research

The integration model, in general, requires validation in order to identify the level of the contribution in the sector with criteria of a more solid nature. This constitutes a valuable contribution that would enable policy management to implement tools in favour of the creation of comprehensive strategies for the reduction of the negative impacts of certain economic and industrial activities, which represent a critical issue for today’s nations [103,104].

With respect to new research, the study of the synergy between and compensation between the economic well-being of farmers, agricultural production, and ecological preservation are considered [11], as are well as the lack of tools, methodologies, and frameworks to manage the increased complexity of agri-food sustainability as a complex adaptive system [32]. Lastly, the development is required of a model that quantifies the level of depletion of resources due to their excessive use.

## Supporting information

S1 Appendix Interaction and influence between the main variables of integral sustainability in the agri-food sector. (DOCX)

## References

[1] Finanzas & I+D+i. Subvenciones para la agricultura en España: El secreto mejor Guardado. In: Finanzas & I+D+i [Internet]. 18 Jul 2023 [cited 25 Sep 2023]. Available: https://finanzasidi.com/2023/07/18/subvenciones-para-la-agricultura-en-espana-que-son-como-se-pueden-solicitar-y-que-beneficios-aportan/.

[2] Cattaneo CA, Bocchicchio AM. Dinámica sociorganizacional en el sistema agroalimentario. Rev Mex Sociol. 2019;1: 7–35.

[3] Poponi S, Arcese G, Ruggieri A, Pacchera F. Value optimisation for the agri-food sector: A circular economy approach. Bus Strategy Environ. 2022. doi: 10.1002/bse.3274

[4] Graham NT, Iyer G, Wild TB, Dolan F, Lamontagne J, Calvin K. Agricultural market integration preserves future global water resources. One Earth. 2023;6: 1235–1245. doi: 10.1016/j.oneear.2023.08.003

[5] Kheir AMS, Elnashar A, Mosad A, Govind A. An improved deep learning procedure for statistical downscaling of climate data. Heliyon. 2023;9. doi: 10.1016/j.heliyon.2023.e18200 37539241 PMC10393634

[6] Organización de las Naciones Unidas para la Alimentación y la Agricultura (FAO). Agricultura Mundial Hacia los Años 2015/2030: Informe Resumido. Food and Agriculture Organization of the United Nations, editor. Agricultura mundial: hacia los años 2015/2030. 2004. Available: https://www.google.com.mx/books/edition/Agricultura_Mundial/qvr—cUhFcUC?hl=es&gbpv=1.

[7] Organización de las Naciones Unidas para la Alimentación y la Agricultura (FAO). Agricultura Familiar en América Latina y el Caribe: Recomendaciones de Política. Salcedo S, Guzmán L, editors. Santiago: Organización de las Naciones Unidas para la Alimentación y la Agricultura; 2014. Available: www.fao.org/publications.

[8] Organización de las Naciones Unidas para la Alimentación y la Agricultura (FAO), Ediciones Mundi-Prensa. The State of the World’s Land and Water Resources for Food and Agriculture. Managing systems at risk. Organización de las Naciones Unidas para la Alimentación y la Agricultura (FAO), Mundi-Prensa, editors. Roma: Mundi-Prensa, Organización de las Naciones Unidas para la Alimentación y la Agricultura (FAO); 2012.

[9] Schneider JM, Zabel F, Schünemann F, Delzeit R, Mauser W. Global cropland could be almost halved: Assessment of land saving potentials under different strategies and implications for agricultural markets. PLoS One. 2022;17. doi: 10.1371/journal.pone.0263063 35192630 PMC8863228

[10] Rodríguez Eugenio N, McLaughlin M, Pennock D. La contaminación del suelo: una realidad oculta. Roma; 2019.

[11] Benabderrazik K, Kopainsky B, Tazi L, Joerin J, Six J. Agricultural intensification can no longer ignore water conservation–A systemic modelling approach to the case of tomato producers in Morocco. Agric Water Manag. 2021;256. doi: 10.1016/j.agwat.2021.107082

[12] Kuhmonen I, Kuhmonen T. Transitions through the dynamics of adaptive cycles: Evolution of the Finnish agrifood system. Agric Syst. 2023;206. doi: 10.1016/j.agsy.2023.103604

[13] Manuel Alonso Cortés. El Análisis de Ciclo de vida y sus principales softwares como herramientas de cálculo. In: Revista Digital Inesem. 20 Oct 2015.

[14] Saxena P, Stavropoulos P, Kechagias J, Salonitis K. Sustainability assessment for manufacturing operations. Energies (Basel). 2020;13. doi: 10.3390/en13112730

[15] de Carvalho Araújo CK, Bigarelli Ferreira M, Salvador R, de Carvalho Araújo CKC, Camargo BS, de Carvalho Araújo Camargo SK, et al. Life cycle assessment as a guide for designing circular business models in the wood panel industry: A critical review. J Clean Prod. 2022;355. doi: 10.1016/j.jclepro.2022.131729

[16] Scarpellini S, Valero-Gil J, Moneva JM, Andreaus M. Environmental management capabilities for a “circular eco-innovation.” Bus Strategy Environ. 2020;29: 1850–1864. doi: 10.1002/bse.2472

[17] Batlles-delaFuente A, Abad-Segura E, González-Zamar MD, Cortés-García FJ. An Evolutionary Approach on the Framework of Circular Economy Applied to Agriculture. Agronomy. 2022;12. doi: 10.3390/agronomy12030620

[18] Xia F, Zhang Z, Zhang Q, Huang H, Zhao X. Life cycle assessment of greenhouse gas emissions for various feedstocks-based biochars as soil amendment. Science of the Total Environment. Elsevier B.V.; 2024. doi: 10.1016/j.scitotenv.2023.168734 38007117

[19] Paniagua S, Lebrero R, Muñoz R. Syngas biomethanation: Current state and future perspectives. Bioresour Technol. 2022;358. doi: 10.1016/j.biortech.2022.127436 35680093

[20] Yang X, Han D, Zhao Y, Li R, Wu Y. Environmental evaluation of a distributed-centralized biomass pyrolysis system: A case study in Shandong, China. Science of the Total Environment. 2020;716. doi: 10.1016/j.scitotenv.2020.136915 32036128

[21] Tsui TH, van Loosdrecht MCM, Dai Y, Tong YW. Machine learning and circular bioeconomy: Building new resource efficiency from diverse waste streams. Bioresource Technology. Elsevier Ltd; 2023. doi: 10.1016/j.biortech.2022.128445 36473583

[22] Dinelli G, Chen Q, Scuderi A, Via G La, Timpanaro G, Sturiale L. The Digital Applications of “Agriculture 4.0”: Strategic Opportunity for the Development of the Italian Citrus Chain. 2022. doi: 10.3390/agriculture

[23] Uppal N, Pappu A, Gowri VKS, Thakur VK. Cellulosic fibres-based epoxy composites: From bioresources to a circular economy. Industrial Crops and Products. Elsevier B.V.; 2022. doi: 10.1016/j.indcrop.2022.114895

[24] De Menna F, Malagnino RA, Vittuari M, Molari G, Seddaiu G, Deligios PA, et al. Potential biogas production from artichoke byproducts in Sardinia, Italy. Energies (Basel). 2016;9. doi: 10.3390/en9020092

[25] De Menna F, Malagnino RA, Vittuari M, Segrè A, Molari G, Deligios PA, et al. Optimization of agricultural biogas supply chains using artichoke byproducts in existing plants. Agric Syst. 2018;165: 137–146. doi: 10.1016/j.agsy.2018.06.008

[26] Cocco D, Deligios PA, Ledda L, Sulas L, Virdis A, Carboni G. LCA study of oleaginous bioenergy chains in a Mediterranean environment. Energies (Basel). 2014;7: 6258–6281. doi: 10.3390/en7106258

[27] Montegiove N, Gambelli AM, Calzoni E, Bertoldi A, Puglia D, Zadra C, et al. Biogas Production with Residuals Deriving from Olive Mill Wastewater and Olive Pomace Wastes: Quantification of Produced Energy, Spent Energy, and Process Efficiency. Agronomy. 2024;14: 531. doi: 10.3390/agronomy14030531

[28] Luqman M, Shahid T, Awan MUF, Kashif SUR, Arooj F, Awan AR. Quantification and characterization of microplastics (MPs) pollution in peri-uburban agricultural lands of Lahore, Pakistan. PLoS One. 2023;18: e0291760. doi: 10.1371/journal.pone.0291760 37788245 PMC10547192

[29] Aznar Sanchez JA, Mendoza JMF, Ingrao C, Failla S, Bezama A, Nemecek T, et al. Indicators for Circular Economy in the Agri-food Sector. Resour Conserv Recycl. 2020;163: 105028. doi: 10.1016/j.resconrec.2020.105028

[30] Rodríguez Aldabe Y. Potenciar la resiliencia de las ciudades y sus territorios de pertenencia en el marco de los acuerdos sobre cambio climático y de la Nueva Agenda Urbana. Santiago; 2018. Available: www.cepal.org/es/suscripciones.

[31] Sheppard P, Garcia-Garcia G, Stone J, Rahimifard S. A complete decision-support infrastructure for food waste valorisation. J Clean Prod. 2020;247. doi: 10.1016/j.jclepro.2019.119608

[32] Riemens M, Sønderskov M, Moonen AC, Storkey J, Kudsk P. An Integrated Weed Management framework: A pan-European perspective. European Journal of Agronomy. 2022;133. doi: 10.1016/j.eja.2021.126443

[33] Ramos TB, Darouich H, Oliveira AR, Farzamian M, Monteiro T, Castanheira N, et al. Water use and soil water balance of Mediterranean tree crops assessed with the SIMDualKc model in orchards of southern Portugal. Agric Water Manag. 2023;279. doi: 10.1016/j.agwat.2023.108209

[34] Zhang CY, Oki T. Water pricing reform for sustainable water resources management in China’s agricultural sector. Agricultural Water Management. Elsevier B.V.; 2023. doi: 10.1016/j.agwat.2022.108045

[35] Secchi MA, Fernandez JA, Stamm MJ, Durrett T, Prasad PVV, Messina CD, et al. Effects of heat and drought on canola (Brassica napus L.) yield, oil, and protein: A meta-analysis. Field Crops Res. 2023;293. doi: 10.1016/j.fcr.2023.108848

[36] Dono G, Cortignani R, Doro L, Giraldo L, Ledda L, Pasqui M, et al. An Integrated Assessment of the Impacts of Changing Climate Variability on Agricultural Productivity and Profitability in an Irrigated Mediterranean Catchment. Water Resources Management. 2013;27: 3607–3622. doi: 10.1007/s11269-013-0367-3

[37] Dossou-Yovo ER, Devkota KP, Akpoti K, Danvi A, Duku C, Zwart SJ. Thirty years of water management research for rice in sub-Saharan Africa: Achievement and perspectives. Field Crops Research. Elsevier B.V.; 2022. doi: 10.1016/j.fcr.2022.108548

[38] Ben Ammar H, Arena D, Treccarichi S, Di Bella MC, Marghali S, Ficcadenti N, et al. The Effect of Water Stress on the Glucosinolate Content and Profile: A Comparative Study on Roots and Leaves of Brassica oleracea L. Crops. Agronomy. 2023;13. doi: 10.3390/agronomy13020579

[39] De Menna F, Malagnino RA, Vittuari M, Segrè A, Molari G, Deligios PA, et al. Optimization of agricultural biogas supply chains using artichoke byproducts in existing plants. Agric Syst. 2018;165: 137–146. doi: 10.1016/j.agsy.2018.06.008

[40] Straffelini E, Tarolli P. Climate change-induced aridity is affecting agriculture in Northeast Italy. Agric Syst. 2023;208. doi: 10.1016/j.agsy.2023.103647

[41] Pan Y, Zhu Y, Lü H, Yagci AL, Fu X, Liu E, et al. Accuracy of agricultural drought indices and analysis of agricultural drought characteristics in China between 2000 and 2019. Agric Water Manag. 2023;283. doi: 10.1016/j.agwat.2023.108305

[42] Chu X Lei, Lu Z, Wei D, Lei G Ping. Effects of land use/cover change (LUCC) on the spatiotemporal variability of precipitation and temperature in the Songnen Plain, China. J Integr Agric. 2022;21: 235–248. doi: 10.1016/S2095-3119(20)63495-5

[43] Pino-Vargas EM, Ascencios DR. Sustainability of olive cultivation under a climatological approach in an arid region at the Atacama Desert. Ciencia Tecnologia Agropecuaria. 2022;23. doi: 10.21930/RCTA.VOL23_NUM3_ART:2652.

[44] Diogo V, Helfenstein J, Mohr F, Varghese V, Debonne N, Levers C, et al. Developing context-specific frameworks for integrated sustainability assessment of agricultural intensity change: An application for Europe. Environ Sci Policy. 2022;137: 128–142. doi: 10.1016/j.envsci.2022.08.014

[45] Cook BR, Satizábal P, Curnow J. Humanising agricultural extension: A review. World Dev. 2021;140. doi: 10.1016/j.worlddev.2020.105337

[46] Allahyari MS, Sadeghzadeh M. Agricultural Extension Systems Toward SDGs 2030: Zero Hunger. Springer. 2020. pp. 41–52. doi: 10.1007/978-3-319-95675-6_2

[47] Vermunt DA, Wojtynia N, Hekkert MP, Van Dijk J, Verburg R, Verweij PA, et al. Five mechanisms blocking the transition towards ‘nature-inclusive’ agriculture: A systemic analysis of Dutch dairy farming. Agric Syst. 2022;195. doi: 10.1016/j.agsy.2021.103280

[48] Howard M, Yan X, Mustafee N, Charnley F, Böhm S, Pascucci S. Going beyond waste reduction: Exploring tools and methods for circular economy adoption in small-medium enterprises. Resour Conserv Recycl. 2022;182. doi: 10.1016/j.resconrec.2022.106345

[49] Trabelsi M, Casprini E, Fiorini N, Zanni L. Unleashing the value of artificial intelligence in the agri-food sector: where are we? British Food Journal. 2023;125: 482–515. doi: 10.1108/BFJ-11-2022-1014

[50] Cañoles M, Valdés O, Rojas L, Galáz JC, Coz F, Díaz N, et al. Estudio de Economía Circular en el Sector Agroalimentario Chileno. Santiago de Chile; 2019 Dec. Available: https://www.odepa.gob.cl/wp-content/uploads/2019/12/EstEconomiaCircular2019.pdf.

[51] Poponi S, Arcese G, Mosconi EM, Di Trifiletti MA. Entrepreneurial drivers for the development of the circular business model: The role of academic spin-Off. Sustainability (Switzerland). MDPI; 2020. doi: 10.3390/su12010423

[52] Congreso nacional del medio ambiente. Retos del sector agroalimentario en los procesos industriales. 2016.

[53] Kiritsis D, Bufardi A, Xirouchakis P. Research issues on product lifecycle management and information tracking using smart embedded systems. Advanced Engineering Informatics. 2003;17: 189–202. doi: 10.1016/j.aei.2004.09.005

[54] Monaga-Reina R, de-Las-Heras A, Luque-Sendra A, Lama-Ruíz JR. Improvement of sustainability management through a plm structure. Good practices and a case study. Dyna (Spain). 2021;96: 373–378. doi: 10.6036/9915

[55] Vila C, Abellán-Nebot J V., Albiñana JC, Hernández G. An Approach to Sustainable Product Lifecycle Management (Green PLM). Procedia Engineering. Elsevier Ltd; 2015. pp. 585–592. doi: 10.1016/j.proeng.2015.12.608

[56] Salonitis K, Stavropoulos P. On the integration of the cax systems towards sustainable production. Procedia CIRP. Elsevier B.V.; 2013. pp. 115–120. doi: 10.1016/j.procir.2013.06.178

[57] Kiritsis D. PLM and Product Embedded Information Devices. IFAC Proceedings Volumes. 2007;40: 8–23. doi: 10.3182/20070523-3-ES-4908.00004

[58] Tseng ML, Chiu ASF, Chien CF, Tan RR. Pathways and barriers to circularity in food systems. Resources, Conservation and Recycling. Elsevier B.V.; 2019. pp. 236–237. doi: 10.1016/j.resconrec.2019.01.015

[59] Krstić M, Agnusdei GP, Miglietta PP, Tadić S. Logistics 4.0 toward circular economy in the agri-food sector. Sustainable Futures. 2022;4. doi: 10.1016/j.sftr.2022.100097

[60] Fiore M, Mongiello M. Blockchain Technology to Support Agri-Food Supply Chains: A Comprehensive Review. IEEE Acces. 2016;4: 1–14. doi: 10.1109/ACCESS.2017.DOI

[61] Muñoz-Ulecia E, Bernués A, Briones-Hidrovo A, Casasús I, Martín-Collado D. Dependence on the socio-economic system impairs the sustainability of pasture-based animal agriculture. Sci Rep. 2023;13. doi: 10.1038/s41598-023-41524-4 37653233 PMC10471625

[62] de Cunzo F, Petri A, Zaccaria A, Sbardella A. The trickle down from environmental innovation to productive complexity. Sci Rep. 2022;12. doi: 10.1038/s41598-022-25940-6 36550185 PMC9780348

[63] Poponi S, Arcese G, Pacchera F, Martucci O. Evaluating the transition to the circular economy in the agri-food sector: Selection of indicators. Resour Conserv Recycl. 2022;176. doi: 10.1016/j.resconrec.2021.105916

[64] Schöggl JP, Stumpf L, Baumgartner RJ. The narrative of sustainability and circular economy ‐ A longitudinal review of two decades of research. Resources, Conservation and Recycling. Elsevier B.V.; 2020. doi: 10.1016/j.resconrec.2020.105073

[65] Pesce M, Tamai I, Guo D, Critto A, Brombal D, Wang X, et al. Circular economy in China: Translating principles into practice. Sustainability (Switzerland). 2020;12. doi: 10.3390/su12030832

[66] Collado AD. La agricultura del futuro: cambios y desafíos. In: La agricultura del futuro: cambios y desafíos.

[67] Crippa M, Solazzo E, Guizzardi D, Monforti-Ferrario F, Tubiello FN, Leip A. Food systems are responsible for a third of global anthropogenic GHG emissions. Nat Food. 2021;2: 198–209. doi: 10.1038/s43016-021-00225-9 37117443

[68] Ramírez-Cuesta JM, Intrigliolo DS, Lorite IJ, Moreno MA, Vanella D, Ballesteros R, et al. Determining grapevine water use under different sustainable agronomic practices using METRIC-UAV surface energy balance model. Agric Water Manag. 2023;281. doi: 10.1016/j.agwat.2023.108247

[69] Calafat-Marzal C, Sánchez-García M, Marti L, Puertas R. Agri-food 4.0: Drivers and links to innovation and eco-innovation. Comput Electron Agric. 2023;207. doi: 10.1016/j.compag.2023.107700

[70] Cesco S, Sambo P, Borin M, Basso B, Orzes G, Mazzetto F. Smart agriculture and digital twins: Applications and challenges in a vision of sustainability. European Journal of Agronomy. 2023;146. doi: 10.1016/j.eja.2023.126809

[71] Nabavi-Pelesaraei A, Ghasemi-Mobtaker H, Salehi M, Rafiee S, Chau KW, Ebrahimi R. Machine Learning Models of Exergoenvironmental Damages and Emissions Social Cost for Mushroom Production. Agronomy. 2023;13. doi: 10.3390/agronomy13030737

[72] Ma W, Liu T, Li W, Yang H. The role of agricultural machinery in improving green grain productivity in China: Towards trans-regional operation and low-carbon practices. Heliyon. 2023;9. doi: 10.1016/j.heliyon.2023.e20279 37767503 PMC10520308

[73] Mies A, Gold S. Mapping the social dimension of the circular economy. Journal of Cleaner Production. Elsevier Ltd; 2021. doi: 10.1016/j.jclepro.2021.128960

[74] Bassi AM, Bianchi M, Guzzetti M, Pallaske G, Tapia C. Improving the understanding of circular economy potential at territorial level using systems thinking. Sustain Prod Consum. 2021;27: 128–140. doi: 10.1016/j.spc.2020.10.028

[75] Nyam YS, Kotir JH, Jordaan AJ, Ogundeji AA, Adetoro AA, Orimoloye IR. Towards understanding and sustaining natural resource systems through the systems perspective: A systematic evaluation. Sustainability (Switzerland). 2020;12: 1–20. doi: 10.3390/su12239871

[76] Dianat H, Wilkinson S, Williams P, Khatibi H. Planning the resilient city: Investigations into using “causal loop diagram” in combination with “UNISDR scorecard” for making cities more resilient. International Journal of Disaster Risk Reduction. 2021;65. doi: 10.1016/j.ijdrr.2021.102561

[77] Sterman John. Business dynamics: systems thinking and modeling for a complex world. Irwin/McGraw-Hill; 2000.

[78] León Romero LP, Aguilar Fernández M, Francisco Márquez M, Zamora Polo F, Luque Sendra A. Characterization of the Information System Integrated to the Construction Project Management Systems. SSRN. 2023. 10.2139/ssrn.4507812. PMC11152955 38841493

[79] Checkland P, Poulter J. Soft Systems Methodology. Método Radical para Integrar Actividades Organizativas. Milrazones, editor. 2010.

[80] Checkland P, Scholes J. La Metodología de los Sistemas Suaves de Acción. Grupo Noriega, editor. Ciudad de México; 1994.

[81] Cummings S, Hassard J, Rowlinson M, Brocklesby J, Davies J. Demystifying the viable System Model as a Tool for Organisational Analysis The end goal of management? View project A New History of Management (with. Article in Asia Pacific Journal of Operational Research. 1995. Available: https://www.researchgate.net/publication/266311839.

[82] Aguilar Fernández M, Patiño Ortiz J, Jarquín BG, Fortanell Estrada P, Cedillo JAÁ. The Viable Systems Model: a Literature Review. International Journal of Latest Research in Science and Technology. 2021;10: 34–36. doi: 10.29111/ijlrst-2019-10968

[83] Kaplan RS, Norton DP. The Balanced Scoredcard: Translating Stratey into Action. 2nd ed. Gestión 2000, editor. Barcelona: Harvard Business School Press; 2002. Available: www.FreeLibros.me.

[84] Loewy T. El enfoque sistémico como criterio operativo y geográfico: La sostenibilidad agrícola. Estudios económicos. 2021;38: 83–98. doi: 10.52292/j.estudecon.2021.2300

[85] Zhang X, Wu K-S, He M. Concave-convex effect of financial resilience on corporate financial performance: quantile regression approach. Humanit Soc Sci Commun. 2023;10: 654. doi: 10.1057/s41599-023-02169-w

[86] Sharma R, Kamble SS, Gunasekaran A, Kumar V, Kumar A. A systematic literature review on machine learning applications for sustainable agriculture supply chain performance. Comput Oper Res. 2020;119. doi: 10.1016/j.cor.2020.104926

[87] Ulukan D, Bergkvist G, Lana M, Fasse A, Mager G, Öborn I, et al. Combining sustainable livelihood and farm sustainability approaches to identify relevant intensification options: Implications for households with crop-based and gathering-based livelihoods in Tanzania. Ecol Indic. 2022;144. doi: 10.1016/j.ecolind.2022.109518

[88] Rossignoli CM, Manyise T, Shikuku KM, Nasr-Allah AM, Dompreh EB, Henriksson PJG, et al. Tilapia aquaculture systems in Egypt: Characteristics, sustainability outcomes and entry points for sustainable aquatic food systems. Aquaculture. 2023;577. doi: 10.1016/j.aquaculture.2023.739952

[89] Piccoli I, Grillo F, Longo M, Furlanetto I, Ragazzi F, Obber S, et al. A farm-scale sustainability assessment of the anaerobic digestate application methods. European Journal of Agronomy. 2023;146. doi: 10.1016/j.eja.2023.126811

[90] Robling H, Abu Hatab A, Säll S, Hansson H. Measuring sustainability at farm level–A critical view on data and indicators. Environmental and Sustainability Indicators. 2023;18. doi: 10.1016/j.indic.2023.100258

[91] Sarkodie SA, Owusu PA. Assessment of global fish footprint reveals growing challenges for sustainable production and consumption. Mar Pollut Bull. 2023;194. doi: 10.1016/j.marpolbul.2023.115369 37556861

[92] Tomar A. Sustainable photovoltaic based protective environment controlled farming technology as economy boosters for agro-sectors. Smart Agricultural Technology. 2023;5. doi: 10.1016/j.atech.2023.100237

[93] Nurgazina J, Pakdeetrakulwong U, Moser T, Reiner G. Distributed ledger technology applications in food supply chains: A review of challenges and future research directions. Sustainability (Switzerland). MDPI AG; 2021. doi: 10.3390/su13084206

[94] Manzoor S, Dar AH, Dash KK, Pandey VK, Srivastava S, Bashir I, et al. Carbon dots applications for development of sustainable technologies for food safety: A comprehensive review. Applied Food Research. 2023;3. doi: 10.1016/j.afres.2023.100263

[95] Acosta Flores J. Ingeniería de sistemas. Un enfoque interdisciplinario. 2nd ed. Alfaomega Grupo Editor S de CM, editor. Ciudad de Meéxico: Alfaomega Grupo Editor, SA de CV México; 2017.

[96] Farissi A, El Oumami M, Beidouri Z. Moroccan Agro-Food Companies: Performance Evaluation through the Balanced Scorecard Method. Int J Sup Chain Mgt. 2020. Available: http://excelingtech.co.uk/.

[97] Verdecho MJ, Pérez Perales D, Alarcón Valero F. Proposal of a Customer-Oriented Sustainable Balanced Scorecard for Agri-Food Supply Chains. In: Springer C, editor. Springer. Springer, Cham; 2020. pp. 233–240. 10.1007/978-3-030-44530-0_28.

[98] Yu. N K, A.V. U. Specific Features in Management of Agro-Food System Development Chair of Information support and modeling of economic systems in agriculture. Advances in Economics, Business and Management Research. 2020;147: 380–384.

[99] Maturana H, Varela F. De Maquinas Y Seres Vivos: Autopoiesis: La Organización De Lo Vivo. 6th ed. Lumen México, editor. Lumen México; 2004.

[100] Ross A. An Introduction to Cybernetics. 2nd ed. Martino Fine Books, editor. Martino Fine Books; 2015.

[101] Stafford Beer. Diagnosing the System for Organizations. 1st ed. Wiley, editor. Wiley; 1985.

[102] Wiener N. Cybernetics. Or the Control and Communication in the Animal and the Machine. 2nd ed. Martino Fine Books, editor. Martino Fine Books; 2013.

[103] Hjorth T, Huseinovic E, Hallström E, Strid A, Johansson I, Lindahl B, et al. Changes in dietary carbon footprint over ten years relative to individual characteristics and food intake in the Västerbotten Intervention Programme. Sci Rep. 2020;10. doi: 10.1038/s41598-019-56924-8 31913331 PMC6949226

[104] Azimi MN, Rahman MM, Nghiem S. Linking governance with environmental quality: a global perspective. Sci Rep. 2023;13. doi: 10.1038/s41598-023-42221-y 37699950 PMC10497530

