# Supplementary material for: Characterisation and causal model of the holistic dynamics of the integral sustainability of the agri-food system
Source: PLoS One. 2024 Jun 27;19(6):e0305743. doi: 10.1371/journal.pone.0305743 (PMC11210858; doi:10.1371/journal.pone.0305743)
Supplement: S1 Appendix — (DOCX) [file pone.0305743.s001.docx]

**Characterization and Causal Model of the Holistic Dynamics of the Integral Sustainability of the Agrifood System**

Leon-Romero Luvis P. ^1,2*^, Zamora-Polo Francisco ^1^, Luque-Sendra Amalia ^1^, Aguilar-Fernández Mario ^2^, Francisco-Márquez Misaela ^2^

^1^ Universidad de Sevilla, Departamento de Ingeniería del Diseño. Escuela Politécnica Superior. Virgen de África, 7. 41011 Sevilla, España

^2^ Instituto Politécnico Nacional, Unidad Profesional Interdisciplinaria de Ingeniería y Ciencias Sociales y Administrativas, Av. Té 950, Col. Granjas México, CP. 08400, México City, México

**Correspondence:** Luvis P. León Romero Email: lleonr2100@alumno.ipn.mx

**S1 APPENDIX 1**

**Interaction and influence between the main variables of integral sustainability in the agri-food sector**

The present document is intended to explain the methodology used, which is the authors' own, within the framework of the review of the literature consulted mainly on the Web of Science and Scopus.

The table 1 shows the dynamic hypotheses used in the study, starting from an initial one traditionally known in the sustainability cycle given by the linkage of economic, social and environmental variables cycle 1.

Cycle 2 represents dynamic hypothesis 1 where process variables are integrated into the traditional ones.

Cycle 3 represents dynamic hypothesis 2 where technology variables are integrated into the traditional ones.

Finally, cycle 4 integrates both process and technology variables to the traditional ones, having a more integral interaction in the system under study.

**Table 1 Dynamic Hypotheses and system interactions**

| **Dynamic Hypotheses** | **Parameter** | **System Interaction** |
| --- | --- | --- |
| **Cicle 1** | Traditional model (Economy + environment + society) | -Creation of strategies to strengthen economic, social, and environmental links to guarantee future resources. |
| **Cicle 2** | Process + traditional model | -Specific controls on waste generating organizations. -Qualified procurement for efficient operations. -Reduction of costs and establishment of competitive market prices. -Selection of raw materials suitable for the process and the environment. -Generation of jobs with quality guarantees. [1,2] |
| **Cicle 3** | Technology + traditional model | -Encourages innovation.  -Encourages the development of society.  -Facilitates mechanisms that reduce environmental impacts.  -Data and information management to support decisions.  -Effort reduction. [3–5] |
| **Cicle 4** | Process + Technplogy + traditional model | -Improved transactions. -Security in processes. -Continuous monitoring of operations. -Reliable information for decision-making. -Use of eco-friendly and user-friendly tools, materials, and methods. -Improved process flexibility and performance. -Trained staff to address issues [6–9] |

**Level of interaction and influence between the main variables of integral sustainability in the agri-food sector**

The most commonly used sustainability variables or dimensions represent economy, environment and society, as evidenced in [9–17]. However, using this study, the following is annexed to this model: production and technology, therefore creating the so-called Pentagon of sustainability, as represented in Fig. 1.

**
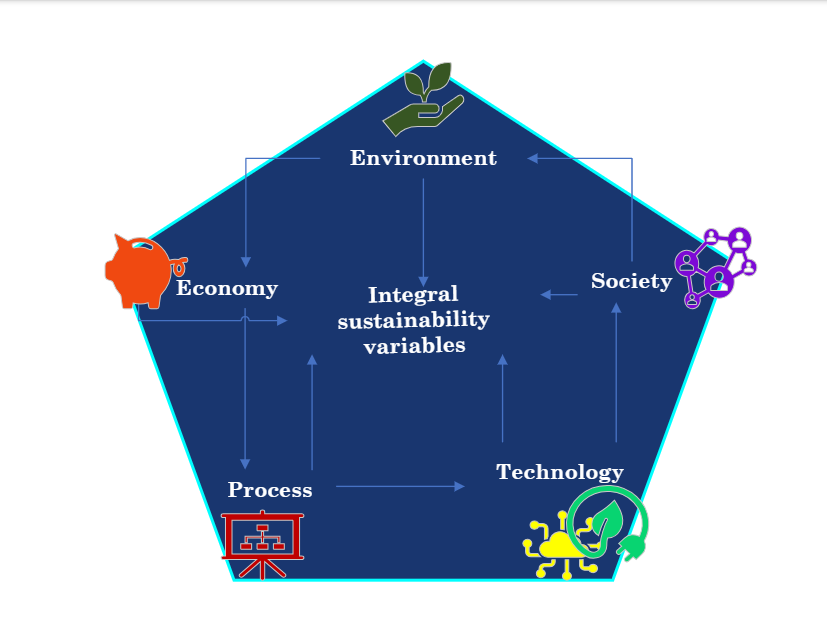
**

**Fig. 1 Integral sustainability pentagon**

Consider some internal variables, as shown in Fig. 2.

**Fig. 2 Variables of the integral sustainability of the agro-industrial sector**

**Objective**

Reinforce the sustainable structure by integrating variables that guarantee the supremacy and optimization of the use of resources, recognizing their importance and impact by the current economic and environmental crisis.

**Justification**

The methods and productive actions implemented, as well as the use and level of technologies applied, considerably affect the patterns of ecosystems according to their management, depleting resources or prolonging their useful life, which is of interest to explore since the aim is to avoid or slow down the extinction processes of certain reserves; therefore, it is necessary to incorporate variables that directly influence these dynamics [18–24].

**Methodology**

Phase 1: listing of system variables, Phase 2: description of relationships between system variables, and Phase 3: identification of key variables and their categories and interpretation.

**Results**

**Table 2 List of variables to study**

| **Environment** | **Society** | **Process** | **Technology** | **Economy** |
| --- | --- | --- | --- | --- |

**Table 3 Qualitative effect between variables**

| **Integral Sustainability Variables** | **Process** | **Technology** | **Environment** | **Society** | **Economy** |
| --- | --- | --- | --- | --- | --- |
| **Process** | Efficiency  Productivity  Performance. | It requires technology and encourages innovation for its development to contribute to efficient performance. | Emission of greenhouse gases, better use of water resources, soil degradation (extinguishes resources, rendering them worthless for future events) [15,25–32] | Provide opportunities for integration through interdisciplinarity within the framework of employability with quality.  (direct-indirect/local or external contracting)  [12,15,29,30,33] | Fair and affordable prices Investor-friendly profits  investment level according to performance integration of productive sectors [5,15,34] |
| **Technology** | Generate agility in the processes.  Optimal use of resources  Avoid waste [9,31,35–41] | Ease of integration and achievement of goals. | Promote the consumption and rational use of resources, avoiding their deterioration or extinction [9,31] | Facilitates communications and relationships [9,37,38] | Streamlines transactions [9,31,36] |
| **Environment** | Soil and water quality interfere with the yield and efficiency.  Availability of naturally occurring renewable and nonrenewable resources [34,42] | Need for mechanisms to clean, purify, and guarantee safe extractions. | Quality: air, water, land, availability of renewable and nonrenewable resources. | Air quality, water quality, safe disease-free environment [10,12,32,43,44] | Need for investment to remain stable and favorable [42,45] |
| **Society** | Professionalization for the optimal development of activities, sociocultural influence, and scale of consumption of goods and services produced [32,34,46] | Need for technology readiness [24,38] | Adequate use of resources to ensure their future availability [32,35] | Integration and participation, inclusive and resilient. | Opportunities for accessibility to services and products [12,44] |
| **Economy** | Cost of inputs, acquisition of credit, cost of taxes and tariffs, licenses, international stock exchange movements [47] | Procurement for development [48] | Grant investments for improvement and maintenance [31,35,47,49,50] | Enable resources to purchase goods and services (drives the economy itself)[34,44,48] | Development opportunities for everyone. |

| **Very low** | **Low** | **Medium** | **High** | **Very high** |
| --- | --- | --- | --- | --- |
| **1** | **2** | **3** | **4** | **5** |

**Table 4 Quantitative effect between variables**

| **Integral Sustainability Variables** | **Process** | **Technology** | **Environment** | **Society** | **Economy** | **Variable that most affects** |
| --- | --- | --- | --- | --- | --- | --- |
| **Process** | 5 | 4 | 5 | 4 | 4 | **22** |
| **Technology** | 5 | 5 | 4 | 4 | 4 | **22** |
| **Environment** | 5 | 2 | 5 | 3 | 2 | **17** |
| **Society** | 4 | 3 | 3 | 5 | 4 | **19** |
| **Economy** | 4 | 4 | 2 | 3 | 5 | **18** |
| **Variable that is most affected** | **23** | **18** | **19** | **19** | **19** |  |

**Conclusion**

Establishing need in a variable indicates the level of dependence on the other variable, requiring contributions from it but not providing them. Put differently, it is critical for that variable to advance or evolve but does not promote a significant effect for the other variable. For example, it is important to have a healthy environment so as not to affect the quality of life or the health of society. However, the environment does not need highly specialized practices and society for its conservation because it requires more than the participation of society; other sectors would have to be linked since society uniquely would not cause a significant effect.

The matrix is filled in according to the answer to the question: is variable X (yellow column) important for the progress or development of variable Y (blue row)?

The oblique line provides a qualifier of five, extensively corresponding to intersections between the same variables.

**References**

1. Mies A, Gold S. Mapping the social dimension of the circular economy. Journal of Cleaner Production. Elsevier Ltd; 2021. doi:10.1016/j.jclepro.2021.128960

2. Bassi AM, Bianchi M, Guzzetti M, Pallaske G, Tapia C. Improving the understanding of circular economy potential at territorial level using systems thinking. Sustain Prod Consum. 2021;27: 128–140. doi:10.1016/j.spc.2020.10.028

3. Hackfort S. Unlocking sustainability? The power of corporate lock-ins and how they shape digital agriculture in Germany. J Rural Stud. 2023;101. doi:10.1016/j.jrurstud.2023.103065

4. Kheir AMS, Elnashar A, Mosad A, Govind A. An improved deep learning procedure for statistical downscaling of climate data. Heliyon. 2023;9. doi:10.1016/j.heliyon.2023.e18200

5. Zhang X, Wu K-S, He M. Concave-convex effect of financial resilience on corporate financial performance: quantile regression approach. Humanit Soc Sci Commun. 2023;10: 654. doi:10.1057/s41599-023-02169-w

6. Krstić M, Agnusdei GP, Miglietta PP, Tadić S. Logistics 4.0 toward circular economy in the agri-food sector. Sustainable Futures. 2022;4. doi:10.1016/j.sftr.2022.100097

7. Manuel Alonso Cortés. El Análisis de Ciclo de vida y sus principales softwares como herramientas de cálculo. In: Revista Digital Inesem. 20 Oct 2015.

8. de Carvalho Araújo CK, Bigarelli Ferreira M, Salvador R, de Carvalho Araújo CKC, Camargo BS, de Carvalho Araújo Camargo SK, et al. Life cycle assessment as a guide for designing circular business models in the wood panel industry: A critical review. J Clean Prod. 2022;355. doi:10.1016/j.jclepro.2022.131729

9. Sharma R, Kamble SS, Gunasekaran A, Kumar V, Kumar A. A systematic literature review on machine learning applications for sustainable agriculture supply chain performance. Comput Oper Res. 2020;119. doi:10.1016/j.cor.2020.104926

10. Kaklauskas A, Kaklauskiene L. Analysis of the impact of success on three dimensions of sustainability in 173 countries. Sci Rep. 2022;12. doi:10.1038/s41598-022-19131-6

11. Wang Z, Liang F, Lin S-H. Can socially sustainable development be achieved through homestead withdrawal? A hybrid multiple-attributes decision analysis. Humanit Soc Sci Commun. 2023;10. doi:10.1057/s41599-023-02035-9

12. Ulukan D, Bergkvist G, Lana M, Fasse A, Mager G, Öborn I, et al. Combining sustainable livelihood and farm sustainability approaches to identify relevant intensification options: Implications for households with crop-based and gathering-based livelihoods in Tanzania. Ecol Indic. 2022;144. doi:10.1016/j.ecolind.2022.109518

13. Vargas-Santander KG, Álvarez-Diez S, Baixauli-Soler S, Belda-Ruiz M. Developing a country’s sustainability indicator: An analysis of the effect on trade openness. Environmental and Sustainability Indicators. 2023;19. doi:10.1016/j.indic.2023.100280

14. Huang R. SDG-oriented sustainability assessment for Central and Eastern European countries. Environmental and Sustainability Indicators. 2023;19. doi:10.1016/j.indic.2023.100268

15. Rossignoli CM, Manyise T, Shikuku KM, Nasr-Allah AM, Dompreh EB, Henriksson PJG, et al. Tilapia aquaculture systems in Egypt: Characteristics, sustainability outcomes and entry points for sustainable aquatic food systems. Aquaculture. 2023;577. doi:10.1016/j.aquaculture.2023.739952

16. Cordella M, Horn R, Hong SH, Bianchi M, Isasa M, Harmens R, et al. Addressing sustainable development goals in life cycle sustainable assessment: Synergies, challenges and needs. J Clean Prod. 2023;415. doi:10.1016/j.jclepro.2023.137719

17. Piwowar-Sulej K, Iqbal Q. Leadership styles and sustainable performance: A systematic literature review. J Clean Prod. 2023;382. doi:10.1016/j.jclepro.2022.134600

18. Pitkänen K, Karppinen TKM, Kautto P, Pirtonen H, Salmenperä H, Savolahti H, et al. How to measure the social sustainability of the circular economy? Developing and piloting social circular economy indicators in Finland. J Clean Prod. 2023;392. doi:10.1016/j.jclepro.2023.136238

19. Akinyi DP, Ng’ang’a SK, Ngigi M, Mathenge M, Girvetz E. Cost-benefit analysis of prioritized climate-smart agricultural practices among smallholder farmers: evidence from selected value chains across sub-Saharan Africa. Heliyon. 2022;8. doi:10.1016/j.heliyon.2022.e09228

20. MAO H, QUAN Y rong, FU Y. Risk preferences and the low-carbon agricultural technology adoption: Evidence from rice production in China. J Integr Agric. 2023;22: 2577–2590. doi:10.1016/j.jia.2023.07.002

21. Shi H, Kang Y, Ali MAS, Fan H. The influence of internet use on residents’ ecological conservation behaviors: Evidence from Taibai Mountain Nature Reserve, China. Glob Ecol Conserv. 2023;46. doi:10.1016/j.gecco.2023.e02558

22. LI R, CHAI S xi, CHAI Y wei, LI Y wei, CHANG L, CHENG H bo. Straw strip mulching: A sustainable technology for saving water and improving efficiency in dryland winter wheat production. J Integr Agric. 2022;21: 3556–3568. doi:10.1016/j.jia.2022.08.098

23. Zegeye MB, Meshesha GB, Shah MI. Measuring the poverty reduction effects of adopting agricultural technologies in rural Ethiopia: findings from an endogenous switching regression approach. Heliyon. 2022;8. doi:10.1016/j.heliyon.2022.e09495

24. YUE M, LI W jing, JIN S, CHEN J, CHANG Q, Glyn J, et al. Farmers’ precision pesticide technology adoption and its influencing factors: Evidence from apple production areas in China. J Integr Agric. 2023;22: 292–305. doi:10.1016/j.jia.2022.11.002

25. Chakraborti R, Davis KF, DeFries R, Rao ND, Joseph J, Ghosh S. Crop switching for water sustainability in India’s food bowl yields co-benefits for food security and farmers’ profits. Nature Water. 2023. doi:10.1038/s44221-023-00135-z

26. Yang J, Kang S, Chen D, Zhao L, Ji Z, Duan K, et al. South Asian black carbon is threatening the water sustainability of the Asian Water Tower. Nat Commun. 2022;13. doi:10.1038/s41467-022-35128-1

27. Piccoli I, Grillo F, Longo M, Furlanetto I, Ragazzi F, Obber S, et al. A farm-scale sustainability assessment of the anaerobic digestate application methods. European Journal of Agronomy. 2023;146. doi:10.1016/j.eja.2023.126811

28. Montanaro G, Doupis G, Kourgialas N, Markakis E, Kavroulakis N, Psarras G, et al. Management options influence seasonal CO2 soil emissions in Mediterranean olive ecosystems. European Journal of Agronomy. 2023;146. doi:10.1016/j.eja.2023.126815

29. Han G, Niles MT. An adoption spectrum for sustainable agriculture practices: A new framework applied to cover crop adoption. Agric Syst. 2023;212. doi:10.1016/j.agsy.2023.103771

30. Robling H, Abu Hatab A, Säll S, Hansson H. Measuring sustainability at farm level – A critical view on data and indicators. Environmental and Sustainability Indicators. 2023;18. doi:10.1016/j.indic.2023.100258

31. Nti EK, Kranjac-Berisavljevic G, Doke DA, Wongnaa CA, Attafuah EE, Gyan MA. The impact of artisanal gold mining on the sustainability of Ghana’s river basins: The case of the Pra basin. Environmental and Sustainability Indicators. 2023;19. doi:10.1016/j.indic.2023.100264

32. Tourtelier C, Gorman M, Tracy S. Influence of gender on the development of sustainable agriculture in France. J Rural Stud. 2023;101. doi:10.1016/j.jrurstud.2023.103068

33. Reidsma P, Accatino F, Appel F, Gavrilescu C, Krupin V, Manevska Tasevska G, et al. Alternative systems and strategies to improve future sustainability and resilience of farming systems across Europe: from adaptation to transformation. Land use policy. 2023;134. doi:10.1016/j.landusepol.2023.106881

34. Sarkodie SA, Owusu PA. Assessment of global fish footprint reveals growing challenges for sustainable production and consumption. Mar Pollut Bull. 2023;194. doi:10.1016/j.marpolbul.2023.115369

35. Ankrah DA, Anum R, Anaglo JN, Boateng SD. Influence of sustainable livelihood capital on climate variability adaptation strategies. Environmental and Sustainability Indicators. 2023;18. doi:10.1016/j.indic.2023.100233

36. Tomar A. Sustainable photovoltaic based protective environment controlled farming technology as economy boosters for agro-sectors. Smart Agricultural Technology. 2023;5. doi:10.1016/j.atech.2023.100237

37. Dal Mas F, Massaro M, Ndou V, Raguseo E. Blockchain technologies for sustainability in the agrifood sector: A literature review of academic research and business perspectives. Technol Forecast Soc Change. 2023;187. doi:10.1016/j.techfore.2022.122155

38. Nurgazina J, Pakdeetrakulwong U, Moser T, Reiner G. Distributed ledger technology applications in food supply chains: A review of challenges and future research directions. Sustainability (Switzerland). MDPI AG; 2021. doi:10.3390/su13084206

39. Gkogkos G, Lourenço P, Pechlivani EM, Encarnação L, Votis K, Giakoumoglou N, et al. Distributed Ledger Technologies for Food Sustainability indexing. Smart Agricultural Technology. 2023;5: 100312. doi:10.1016/j.atech.2023.100312

40. Manzoor S, Dar AH, Dash KK, Pandey VK, Srivastava S, Bashir I, et al. Carbon dots applications for development of sustainable technologies for food safety: A comprehensive review. Applied Food Research. 2023;3. doi:10.1016/j.afres.2023.100263

41. Singh P, Kaur A. A systematic review of artificial intelligence in agriculture. Deep Learning for Sustainable Agriculture. 2022; 57–80. doi:10.1016/B978-0-323-85214-2.00011-2

42. Malila BP, Kaaya OE, Lusambo LP, Schaffner U, Kilawe CJ. Factors influencing smallholder Farmer’s willingness to adopt sustainable land management practices to control invasive plants in northern Tanzania. Environmental and Sustainability Indicators. 2023;19. doi:10.1016/j.indic.2023.100284

43. Caggiano H, Kocakuşak D, Kumar P, Tier MO. U.S. cities’ integration and evaluation of equity considerations into climate action plans. npj Urban Sustainability. 2023;3. doi:10.1038/s42949-023-00129-6

44. Orsango R, Rajan DS, Senapathy M, Bojago E. An analysis of rural farmers’ livelihood sustainability in Offa district, Southern Ethiopia. J Agric Food Res. 2023;12. doi:10.1016/j.jafr.2023.100610

45. Khan Z, Hossain MR, Badeeb RA, Zhang C. Aggregate and disaggregate impact of natural resources on economic performance: Role of green growth and human capital. Resources Policy. 2023;80. doi:10.1016/j.resourpol.2022.103103

46. Sun G, Lin X, Chen J, Xu N, Xiong P, Li H. Cultural inclusion and corporate sustainability: evidence from food culture and corporate total factor productivity in China. Humanit Soc Sci Commun. 2023;10. doi:10.1057/s41599-023-01649-3

47. Hidayati DR, Garnevska E, Childerhouse P. Enabling sustainable agrifood value chain transformation in developing countries. J Clean Prod. 2023;395. doi:10.1016/j.jclepro.2023.136300

48. Furszyfer Del Rio DD, Sovacool BK, Griffiths S, Foley AM, Furszyfer Del Rio J. A cross-country analysis of sustainability, transport and energy poverty. npj Urban Sustainability. 2023;3. doi:10.1038/s42949-023-00121-0

49. Kong L, Wu T, Xiao Y, Xu W, Zhang X, Daily GC, et al. Natural capital investments in China undermined by reclamation for cropland. Nat Ecol Evol. 2023. doi:10.1038/s41559-023-02198-3

50. Lou J, Hultman N, Patwardhan A, Mintzer I. Corporate motivations and co-benefit valuation in private climate finance investments through voluntary carbon markets. npj Climate Action. 2023;2: 32. doi:10.1038/s44168-023-00063-4
